# Parkinson’s disease severity clustering based on tapping activity on mobile device

**DOI:** 10.1038/s41598-022-06572-2

**Published:** 2022-02-24

**Authors:** Decho Surangsrirat, Panyawut Sri-iesaranusorn, Attawit Chaiyaroj, Peerapon Vateekul, Roongroj Bhidayasiri

**Affiliations:** 1 Assistive Technology and Medical Devices Research Center, National Science and Technology Development Agency, Pathum Thani, Thailand; 2 Mathematical Informatics, Information Science, Nara Institute of Science and Technology, Nara, Japan; 3 Entertainment Technology, School of Information Science, Japan Advanced Institute of Science and Technology, Ishikawa, Japan; 4 Department of Computer Engineering, Faculty of Engineering, Chulalongkorn University, Bangkok, Thailand; 5 Chulalongkorn Centre of Excellence for Parkinson’s Disease and Related Disorders, Department of Medicine, Faculty of Medicine, Chulalongkorn University and King Chulalongkorn Memorial Hospital, Thai Red Cross Society, Bangkok, Thailand; 6 The Academy of Science, The Royal Society of Thailand, Bangkok, Thailand

**Keywords:** Machine learning, Computational neuroscience, Parkinson's disease

## Abstract

In this study, we investigated the relationship between finger tapping tasks on the smartphone and the MDS-UPDRS I–II and PDQ-8 using the mPower dataset. mPower is a mobile application-based study for monitoring key indicators of PD progression and diagnosis. Currently, it is one of the largest, open access, mobile Parkinson’s Disease studies. Data from seven modules with a total of 8,320 participants who provided the data of at least one task were released to the public researcher. The modules comprise demographics, MDS-UPDRS I–II, PDQ-8, memory, tapping, voice, and walking. Finger-tapping is one of the tasks that easy to perform and has been analyzed for the quantitative measurement of PD. Therefore, participants who performed both the tapping activity and MDS-UPDRS I–II rating scale were selected for our analysis. Note that the MDS-UPDRS mPower Survey only contains parts of the original scale and has not been clinimetrically tested for validity and reliability. We obtained a total of 1851 samples that contained the tapping activity and MDS-UPDRS I–II for the analysis. Nine features were selected to represent tapping activity. K-mean was applied as an unsupervised clustering algorithm in our study. For determining the number of clusters, the elbow method, Sihouette score, and Davies–Bouldin index, were employed as supporting evaluation metrics. Based on these metrics and expert opinion, we decide that three clusters were appropriate for our study. The statistical analysis found that the tapping features could separate participants into three severity groups. Each group has different characteristics and could represent different PD severity based on the MDS-UPDRS I–II and PDQ-8 scores. Currently, the severity assessment of a movement disorder is based on clinical observation. Therefore, it is highly dependant on the skills and experiences of the trained movement disorder specialist who performs the procedure. We believe that any additional methods that could potentially assist with quantitative assessment of disease severity, without the need for a clinical visit would be beneficial to both the healthcare professionals and patients.

## Introduction

The severity assessment of a movement disorder is based on clinical observation so is therefore highly dependant on the skills and experiences of the trained movement disorder specialist who performs the procedure. Of the multiple clinical rating scales available for the quantification of neurological disorders, the Hoehn and Yahr rating scale (HY) is the most widely used scale for defining the broad categories of motor function in Parkinson’s disease (PD). It is simple and easy to apply, however, because of its simplicity and lack of detail, the scale is not comprehensive^1^. HY scale is also weighted heavily toward postural instability as the primary index of disease severity, it does not capture impairments from other motor features of PD and gives no information on non-motor problems, thereby leaving other specific aspects unassessed^2^. The Movement Disorder Society’s Unified Parkinson’s Disease Rating Scale (MDS-UPDRS) is also a widely accepted method for assessing disease states in Parkinson’s disease. The test consists of four parts: Part I (non-motor experiences of daily living); Part II (motor experiences of daily living); Part III (motor examination); and Part IV (motor complications). A study by Goetz et al.^3^ shows that the statistical results support the validity of the MDS-UPDRS for rating PD. Martinez et al.^4^ also proposed a relationship between severity level and MDS-UPDRS. Finally, the 8-question Parkinson’s Disease Questionnaire (PDQ-8) is a short version of the 39-question Parkinson’s Disease Questionnaire (PDQ-39), a commonly used PD health-related quality of life questionnaire. A study by Jenkinson et al.^5^ shows that the results from the single index gained from the PDQ-39 are comparable with the PDQ-8. The use of the PDQ-8 is recommended over the PDQ-39 where a shorter form is required and a single index measure of overall health status is acceptable or desirable.

Assess the severity and progression of PD are essential in both clinical practice and research. It could indicate the patient’s disease status, the treatment effect, and alterations in other relevant factors. The methods described earlier consist of rating scales and questionnaires based on the interview and examination or patient self-assessment. However, these evaluations provide estimations of conceptual, non observable factors, and are usually scored on an ordinal scale. Therefore, over the last few years, multiple studies on objective measurements based on the devices capturing physical characteristics of the pathological phenomena have been introduced and investigated^6^. These instrumental evaluations aim to quantify the severity based on statistical data without the bias of the specialist or patient. Huo et al.^7^ developed a sensor system composed of a force-sensor, three inertial measurement units (IMUs), and four custom mechanomyography (MMG) sensors for the quantification of PD. This system was validated with 23 PD patients and 10 healthy participants and could predict the UPDRS scores for the assessment of the bradykinesia, rigidity, and tremor with the 85.4% match on average with physician assessment. Multiple studies have also investigated tremor analysis systems based on signals from IMUs worn around the wrist and finger and the possible use of spiral drawing on a tablet device as a quantitative biomarker of PD^8–12^.

Finger-tapping is one of the tasks that has been analyzed for the quantitative measurement of PD. Roalf et al.^13^ investigated the use of finger-tapping using a highly sensitive light-diode finger tapper for 62 healthy older adults, 131 Alzheimer’s disease patients, 63 PD patients, and 46 mild cognitive impairment patients. Their findings suggest that alterations in tapping patterns are common in their patient groups. Alongside this, Arroyo-Gallego et al.^14^ have presented an algorithm to classify PD from the typing activity on a smartphone. They proposed a set of tapping features based on a covariance, skewness, and kurtosis analysis of the timing information to capture impaired motor signs. The best performing feature achieves 81% for both sensitivity and specificity from 21 PD patients and 23 control participants. Even though the study on finger tapping and PD has been investigated and reported by multiple research teams, most of the studies involve participants in a range of twenty to several hundred PD patients and control participants combined. Therefore, in this study, we investigated the relationship between finger tapping tasks on the smartphone and the MDS-UPDRS I–II and PDQ-8 using the mPower dataset. We would like to explore and validate on a large scale that remote PD severity assessment via a personal smartphone is feasible and could be performed without using any sensitive information. The approach could pave the way to remote anonymous screening and diagnosis in the future.

## Material and methods

### Dataset and feature selection

The mPower dataset, one of the largest, open to researcher access, mobile Parkinson’s Disease study was used in this study^15^. It is a mobile application-based study for monitoring key indicators of PD progression and diagnosis. As a mobile application, the study has been able to survey a large, longitudinal cohort of volunteers with PD and controls. Data from seven modules with a total of 8,320 participants who provided the data of at least one task were released to the public researcher. The modules comprise of demographics, MDS-UPDRS I–II, PDQ-8, memory, tapping, voice, and walking. Over the last few years, the dataset has been analyzed and investigated by many research teams. Schwab et al.^16^ used the combination of walking, voice, tapping, and memory as a digital biomarker for the classification of PD. Their experiments were performed on the data of 1852 participants from the mPower study. Pittman et al.^17^ used the walking module with a total of 3101 unique participants and 35,410 samples to classify participants for the signs of PD. Several other studies focus on the voice module for the classification of PD due to the availability and amount of voice samples included in the dataset^18–20^. There are 5826 unique participants with 65,022 voice samples in the mPower dataset. The tapping module was used in the study by Prince et al.^21^ for the classification of PD based on a convolutional neural network with 949 PD and 866 control participants. Most of the studies on the mPower dataset have the goal of distinguishing between PD and healthy controls.

**Figure 1:**
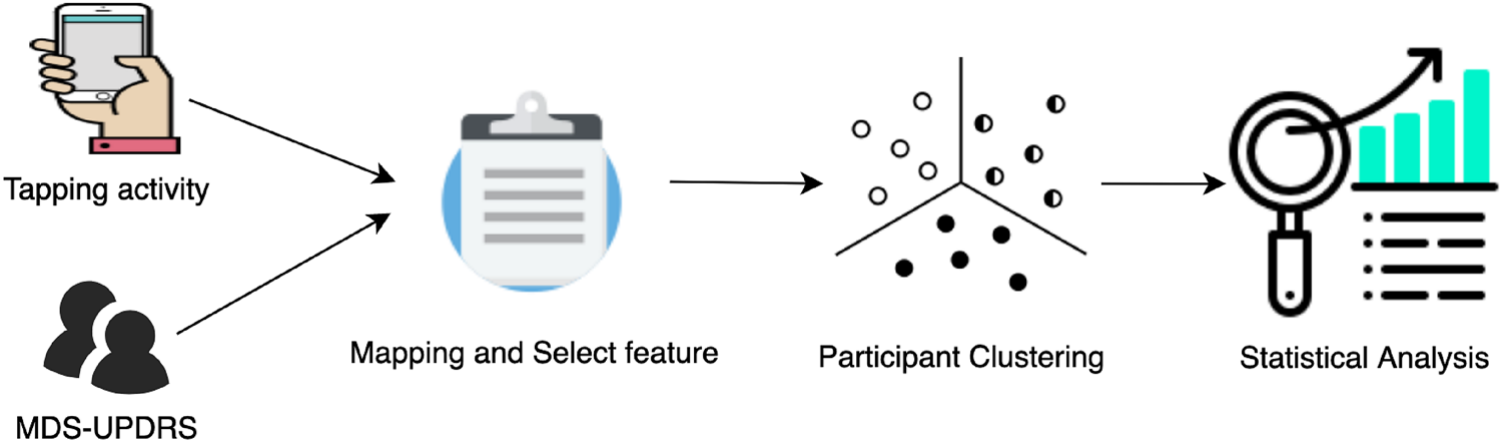
Overview of the process performed in this study.

Participant clustering was performed based on the features selected from the tapping task. Figure 1 summarized the process performed in this study. The tapping activity requires a participant to lay the phone on a flat surface and use two fingers on the same hand to alternatively tap two stationary points on the screen for 20 seconds. For the MDS-UPDRS I–II, a participant needs to respond to the selected questions from Part I and Part II of the MDS-UPDRS which focusing largely on self-evaluation of the motor symptoms of PD; 1.1, 1.3, 1.4, 1.5, 1.7, 1.8, 2.1, 2.4, 2.5, 2.6, 2.7, 2.8, 2.9, 2.10, 2.12, 2.13. PDQ-8 data contains a self-completed Parkinson’s Disease Questionnaire short form. Note that we had been informed by MDS that the MDS-UPDRS mPower Survey has been altered from the original scale and has not been clinimetrically tested for validity and reliability. We acknowledge that the MDS-UPDRS mPower Survey is not a true and actual representation of the MDS-UPDRS and that use of the MDS-UPDRS mPower Survey data in research is not endorsed by MDS.

Tapping activity data consists of 78,887 tasks performed by 8003 unique participants. MDS-UPDRS I–II and PDQ-8 data consist of 2024 and 1334 unique participants, consecutively. To analyze the relationship between activity and questionnaire, the participants were mapped by their participant IDs. Participants who performed both the tapping activity and MDS-UPDRS I–II rating scale were selected for our analysis, with the tapping activity that was recorded closest to the time of the recording of MDS-UPDRS I–II data paired together. Using these parameters, we obtain a total of 1851 samples that contained the tapping activity and MDS-UPDRS I–II for the analysis. 1061 from 1851 samples also contain the PDQ-8 data. The average and standard error of demographic information for the preprocessed dataset is as follows: the age is 44.271 ± 0.436 years, the gender ratio is 0.685 ± 0.011 where 1 is male and 0 is female, the smoker ratio is 0.324 ± 0.011 where 1 is a smoker and 0 is a non-smoker. Other insights include: 52.26% have a college degree, 37.89% have a graduate degree, 82.29% are Caucasian, 4.62% are Latino or Hispanic.

The smartphone records the position and timestamp for every tap on the screen in the tapping activity. The accelerometer data from the smartphone is also recorded to detect if and how the phone was moved during the activity. This information is represented in the dataset in terms of time intervals between taps (*TapInter*), and the positional drifts among taps on the left and right sides (*DriftLeft* and *DriftRight*). For a sequence of timestamps *T*, *TapInter* is defined as the difference between two consecutive taps’ timestamps ($T_t-T_{t-1}$). Positional information is separated into two sequences: $P_{left}$ with *x*-coordinates less than the mean of *x*, and $P_{right}$ greater than or equal to the mean. *DriftLeft* and *DriftRight* are then defined as the Euclidean distances between each consecutive tap positions ($d(P_t,P_{t-1})=\sqrt{(x_t-x_{t-1})^2+(y_t-y_{t-1})^2}$) in $P_{left}$ and $P_{right}$, respectively.

The following features are extracted from all three of these values:

- Basic statistics (min, max, mean, median, range, interquartile range, standard deviation)
- Skewness ($$\frac{\sum _{i=1}^{N}{(x_i - \mu )^3}/N}{s^3}$$; $$s=\sqrt{\sum _{i=1}^{N}{(x_i - \mu )^2}/N}$$).
- Coefficient of variation ($\frac{\sigma }{\mu } \cdot 100$).

Finally, the following features with no direct relations to the aforementioned values are included:

- Total number of taps (*numberTaps*).
- Pairwise Pearson correlation between *x*- and *y*-coordinates of taps (*corXY*).

Similar to prior works that performed data analysis on PD^22–25^, nine features were selected from this list to represent tapping activity in this study:

- *corXY* The correlation between the X- and Y-coordinates of each tap. It represents how the participants’ fingers move, specifically how steep of a diagonal their tapping positions make.
- *numberTaps* The total of number of taps during the tapping activity. It represents how well the participants perform the tapping activity; in the case that the participants’ fingers are jerky, they should be less able to tap properly and thus achieve a lower number of taps.
- *skewDriftRight* The skewness of DriftRight. It represents left or right skew in the distribution of positional drifts among taps on the right side. Positive or negative values indicate that the distribution’s tail is on the right or left side, respectively.
- *skewDriftLeft* The skewness of DriftLeft. It represents left or right skew in the distribution of positional drifts among taps on the left side. Positive or negative values indicate that the distribution’s tail is on the right or left side, respectively.
- *cvTapInter* The coefficient of variation of TapInter. It represents the spread of the time interval between taps. A lower value means more consistently timed taps from the participant. On the other hand, a higher value means less consistent taps.
- *cvDriftRight* The coefficient of variation of DriftRight. It represents the spread of positional drifts among taps on the right side. A lower value means a smaller variation in positional gaps between taps on right side.
- *cvDriftLeft* The coefficient of variation of DriftLeft. It represents the spread of positional drifts among taps on the left side. A lower value means a smaller variation in positional gaps between taps on left side.
- *meanTapInter* The mean of time intervals between taps. A larger value means the participant took longer time on average before making the next successful tap.
- *medianTapInter* The median of time intervals between taps. In the case of a skewed distribution, this value can be used in conjunction with the mean to measure the center of distribution.

To remove the scale bias of the dataset while preserving the shape of dataset, Min–Max Normalization was applied to all nine features to map them into a range of 0–1. The scaling equation used in this study is as follows:

1 $$\begin{aligned} x_{scaled}^{i} = \frac{x^{i}-x_{min}^{i}}{x_{max}^{i}-x_{min}^{i}} \end{aligned}$$

where $x^{i}$ is the original value of feature *i*, $x_{min}^{i}$ and $x_{max}^{i}$ are the minimum value and maximum value of feature *i*, respectively.

### Clustering algorithm

The K-mean clustering algorithm was applied in our study. It is a well-known unsupervised learning algorithm for the uncomplicated data. Countless of prior works such as, Covid-19 analysis^26,27^, disease prediction^28,29^, as well as Parkinson’s disease^30,31^, discovered and introduced the new knowledge in such fields using the algorithm. The concept is to solve the problem of classifying the given data into *k* different clusters through selected parameters and criteria.

We demonstrated the K-mean clustering algorithm and its detail in Algorithm 1. First, we feed the given set of data points and a specific number of cluster *k* as the input. Then, it randomly initializes the centroid of each cluster. The next step is called expectation, which is assign each data point into the closest centroid based on the selected distance function. In this study, we used the Euclidean distance to determine the nearest distance between each data object and the cluster center. After that, the algorithm performs the step named maximization, that is to compute the new centroid of each cluster using the selected criteria such as the mean of cluster. The algorithm repeats both expectation and maximization steps until the centroid position of each cluster does not change. The objective function of the algorithm is the sum of squared error as follows:

2 $$\begin{aligned} E = \sum _{i=1}^{k} \sum _{j=1}^{n_i} \left\| x_{ij} - u_i \right\| ^ 2 \end{aligned}$$

where *k* is the selected number of cluster, $n_i$ is the number of point in cluster *k*, $x_{ij}$ is the *j*-th point in the *i*-th cluster, and $u_i$ is the centroid for the *i*-th cluster. As random initializing the centroid, the algorithm cannot guarantee that the cluster result is the best. To deal with the issue, for each selecting *k*, we perform 1000 randomly different initializing per an experiment, then select the one that satisfies the minimum objective function to reduce the effect of bad initializing. 
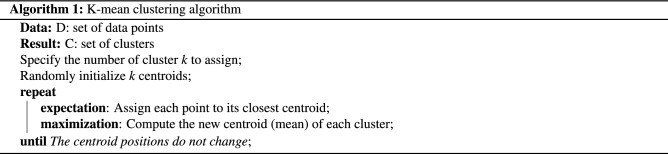


### Evaluation metrics

An elbow method and two additional evaluation metrics, Sihouette and Davies–Bouldin index, were applied for determining the number of clusters in this study. For partitioning clustering, such as K-mean clustering, it is necessary to specify the number of clusters *k* to be generated. However, it is a fundamental issue that the optimal number of clusters is unclear and depends on the method and the parameters used for clustering^32^. One of the solutions we applied to overcome this difficulty is the elbow method which was applied in previous works^33,34^. We also applied the popular and efficient evaluation matrix Silhouette, and Davies–Bouldin index to deal with this issue. Note that, although the optimal number of clusters can be obtained from the evaluation metrics, it is only a guide and the final number of clusters should be considered based on expert interpretation and opinion.

#### Silhouette

The silhouette value was introduced and applied for evaluating the cluster validity^35,36^. It measures the quality of a clustering, that is how similar an object is to its own cluster compared to other clusters. A high silhouette value indicates that the object is well matched to its own cluster and poorly matched to neighboring clusters. If most objects have a high value, then the clustering configuration is appropriate. On the other hand, if there are many points with a low value, then the clustering configuration may have too many or too few clusters. The optimal number of clusters *k* is the one that maximize the average silhouette over a range of selected *k*. Assuming that all of the datapoints have been assigned into *k* clusters, the silhouette score (*s*(*i*)) for a single datapoint *i* is calculated by the mean intra-cluster distance (*a*(*i*)) and the mean nearest-cluster distance (*b*(*i*)) as follow:

3 $$\begin{aligned} a(i)&= \frac{1}{\mid C_i \mid - 1}\sum _{j\in {C_i}, i\ne {j}}^{}{d(i,j)} \end{aligned}$$

4 $$\begin{aligned} b(i)&= \underset{k\ne {i}}{\mathrm {min}} \frac{1}{\mid C_k \mid - 1}\sum _{j\in {C_k}}^{}{d(i,j)} \end{aligned}$$

5 $$\begin{aligned} s(i)&= {\left\{ \begin{array}{ll} 1-a(i)/b(i), & \mathrm {if}\; a(i) < b(i) \\ 0, & \mathrm {if}\; a(i) = b(i) \\ b(i)/a(i)-1, & \mathrm {if}\; a(i) > b(i) \end{array}\right. } \end{aligned}$$

where *d*(*i*, *j*) is the distance between data points *i* and *j* in the cluster $C_{i}$. We divide by $|C_{i}|-1$ because we do not include the distance *d*(*i*, *i*) in the sum. We can interpret *a*(*i*) as a measure of how well *i* assigned to its cluster, while *b*(*i*) represents a neighboring cluster of *i* because it is the next best fit cluster for point *i*.

#### Davies–Bouldin index

Another evaluation metric used in this study is Davies–Bouldin index. It was used in prior works for clustering evaluation^37,38^. This index represents the average similarity measure of each cluster with its most similar cluster, where similarity is the ratio of within-cluster distances ($S_i$) to between-cluster distances ($M_i$). Thus, clusters which are farther apart and less dispersed will result in a better score. Davies–Bouldin index (*DB*) is defined as follow:

6 $$\begin{aligned} S_i&= \left( \frac{1}{T_i} \sum _{j=1}^{T_i}\mid X_j - A_i \mid ^{\;p} \right) ^{1/p} \end{aligned}$$

7 $$\begin{aligned} M_{i,j}&= \; \parallel A_{i} - A_{j} \parallel _{p} \end{aligned}$$

8 $$\begin{aligned} DB&= \frac{1}{N}\sum _{i=1}^{N} \underset{i \ne {j}}{\mathrm {max}}\left( \frac{S_i+S_j}{d_{i,j}}\right) \end{aligned}$$

where $X_j$ is an point assigned to cluster *i*, $A_i$ is the centroid of cluster *i*, and $T_i$ is a number of point belonging to cluster *i*. $\; \parallel A_{i} - A_{j} \parallel _{p}$ is distance between cluster *i* and cluster *j*. The distance of Eqs. (7) and (8) is euclidian in the case that *p* is set to be 2.

### Ethics statement

The studies involving human participants were reviewed and approved by the local Ethics Committees of the Western Institutional Review Board as stated by Bot et al.^15^. Informed consent was obtained from all the participant. Ethical oversight of the study was obtained from Western Institutional Review Board and all methods were carried out in accordance with their guidelines and regulations. Before completing the e-consent process, prospective participants had to pass a five-question quiz evaluating their understanding of the study aims, participant rights, and data sharing options. Their signed consent form was sent to the provided email addresses for verification of their enrollment in the study. There is no additional ethical approval independently for this study. The data contributors allows qualified researchers to analyse the data and publish the findings in open access publications.

## Results and discussion

### Participant clustering

**Figure 2:**
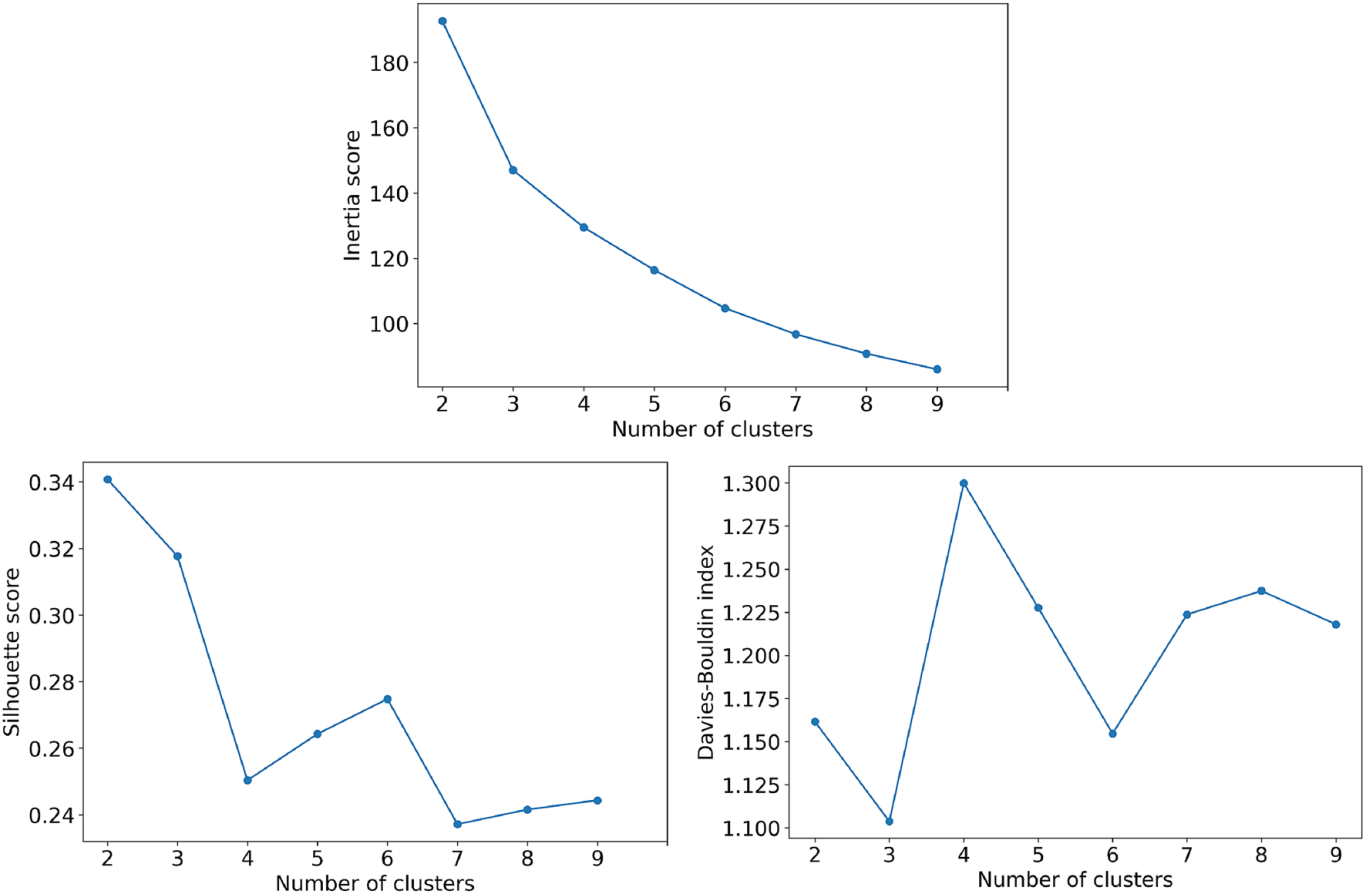
Evaluation results based on Inertia score, Silhouette score, and Davies–Bouldin index.

**Figure 3:**
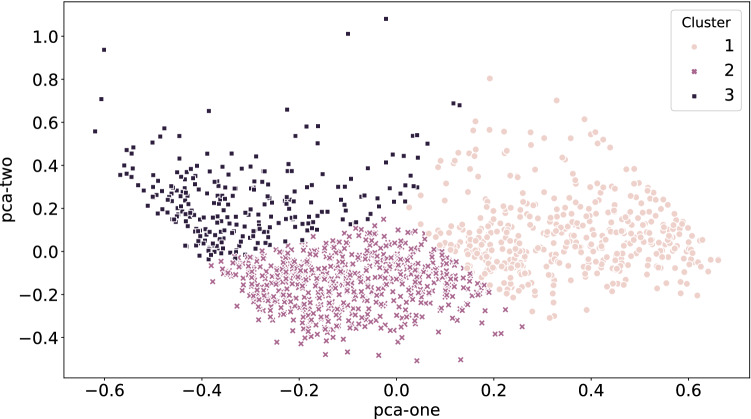
PCA results of the three clusters, each represented by a distinct marker. The number of samples is 594, 897, and 360, for cluster number 1, 2, and 3, respectively. X-axis or pca-one is first rank component with the variance percentage of 50.05, and y-axis or pca-two is the second rank component with the variance percentage of 26.51.

We selected the number of clusters based on the scores of evaluation metrics as shown in Fig. 2. The elbow effect of inertia score and the results of Silhouette and Davies–Bouldin index mostly support the selection of three clusters. While a statistically superior number of clusters could be found at a higher number of clusters, interpreting the clustering results would not be feasible with the number of clusters of 9 or more. Moreover, the trend from clustering evaluation results in Fig. 3 shows that higher *k* might not provide better evaluation scores. Hence, based on the results of evaluation metrics and expert opinion, we decided that three clusters was appropriate for our study.

To investigate the sparseness of the clusters, Fig. 3 illustrates the low-dimensional latent space using principal component analysis (PCA). The figure shows that the clusters can be distinguished clearly in this two-dimensional space. Based on MDS-UPDRS I–II and PDQ-8, cluster one is the lowest severity while cluster three is the highest severity.

**Figure 4:**
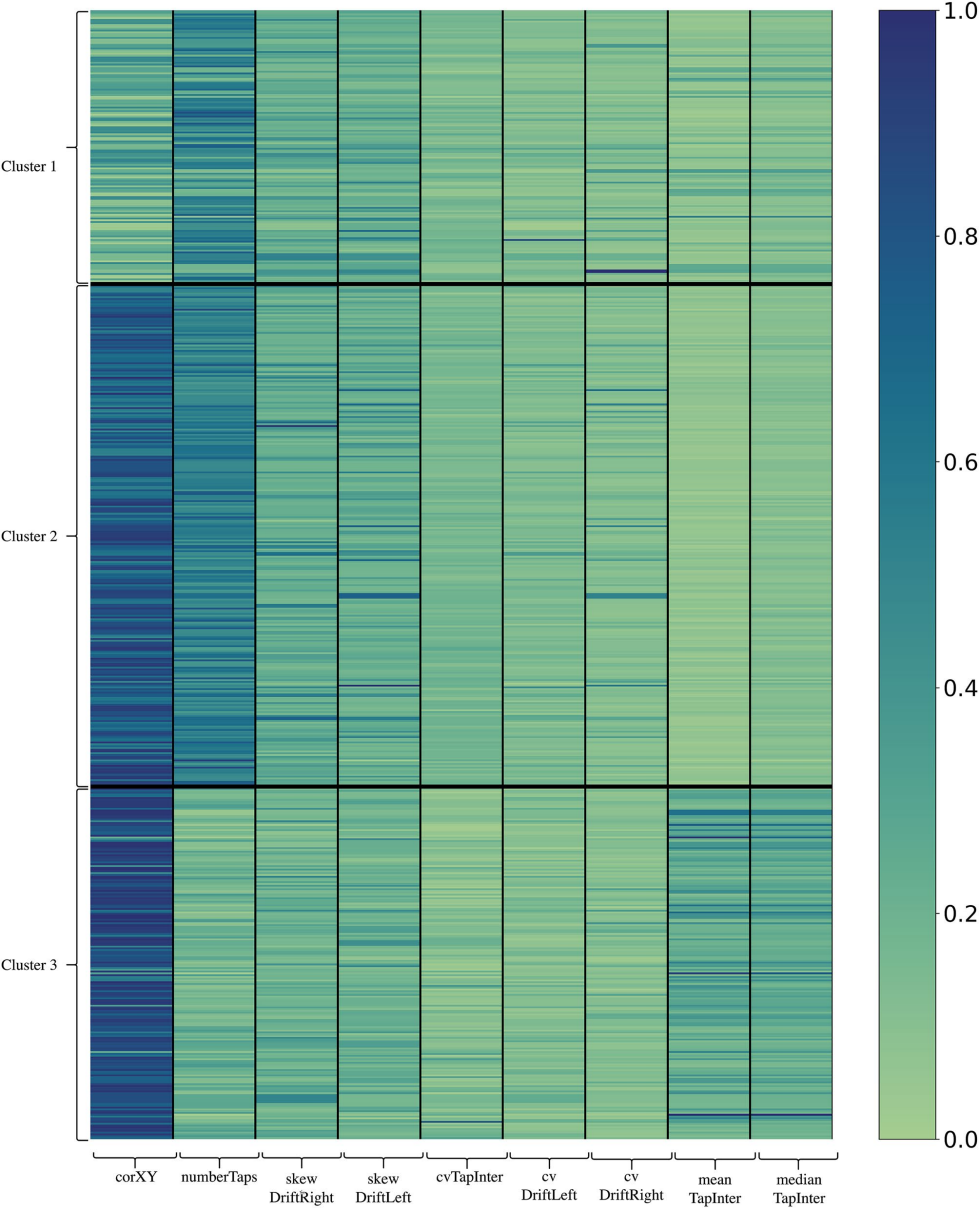
Heatmap plot illustrating the relationship between clusters and features. Each row represents a participant in the cluster. Each column represents the feature used in this study. Blue means high value in that feature for the participant, while green means low value. For example, most of the participants in cluster three have a high value for the corXY feature.

Figure 4 is a heatmap visualization that demonstrates the relationship between each cluster and each feature. We can see that some features can distinguish between clusters. For example, clusters two and three have extremely high values of corXY. However, the value of numberTaps is high for cluster two while the value is low for cluster three. These can imply that corXY and numberTaps play an important factor in the clustering. On the other hand, we cannot visually detect a clear pattern from features such as cvDriftLeft or cvDriftRight.

### Statistical analysis

Table 1 shows MDS-UPDRS I–II and PDQ-8 scores with average and standard error of the mean for the three clusters based on the unsupervised clustering of the tapping features. The samples are evenly distributed between the clusters; 594, 897, and 360, for clusters 1, 2, and 3, respectively. The samples with both MDS-UPDRS I–II and PDQ-8 scores are also more or less evenly distributed; 320, 496, 245 for clusters 1, 2, and 3, respectively. Each cluster can be distinguished by the MDS-UPDRS I–II or PDQ-8 scores, with cluster one being the lowest and cluster three being the highest. Moreover, the score of Part II of the MDS-UPDRS I–II, motor aspects of experiences of daily living (M-EDL), from cluster three is the highest by a significant margin. The experimental results show that the tapping features could be one of the tools for quantification of PD severity based on MDS-UPDRS I–II and PDQ-8 scores.

To illustrate the differences between clusters, we compare our clustering results with the PD severity levels based on MDS-UPDRS scores by Martinez-Martin et al.^4^. The cut-off values for each MDS-UPDRS I–II subscale were determined by the triangulation value of the percentile 90 of the subscale total score, the analysis of receiver operating characteristic (ROC), and the ordinal logistic regression model (OLR). Since the mPower MDS-UPDRS I–II contains part of the original MDS-UPDRS I–II, the score re-scaling was performed. Table 2 shows the percentage of participants in each severity group for the three clusters. For Part I, cluster three had a significantly higher percentage of severe participants. This trend was reiterated for Part II. However, since there is only one participant in Part II in the severe group, cluster three had a significantly higher percentage of moderate participants instead. As for the average score, clusters one and two were assigned to the mild group while cluster three was assigned to the moderate group in Part I. All of the clusters were assigned to the mild group in Part II. The lower scores from the mPower study could be from fact that all of the questions were answered by the patients themselves which could lead to a self-bias. Also, the participants that could be recruited into the study must be able to use a smartphone. Therefore, most moderate or severe PD patients would not be able to participate in the study (Table 3).

**Table 1:** Scores from the mPower MDS-UPDRS I–II and PDQ-8 for the three clusters.

	Cluster one	Cluster two	Cluster three
**mPower MDS-UPDRS I–II**			
Part I (Maximum 24 points)	4.715 ± 0.146	4.645 ± 0.121	5.403 ± 0.215
Part II (Maximum 40 points)	2.603 ± 0.174	3.154 ± 0.166	5.228 ± 0.277
Total (Maximum 64 points)	7.318 ± 0.260	7.799 ± 0.243	10.631 ± 0.421
**PDQ-8**			
Score	8.668 ± 0.759	9.887 ± 0.664	14.405 ± 1.000

**Table 2:** Percentage of participants from the mPower MDS-UPDRS I–II in each severity group for the three clusters.

	Cluster one	Cluster two	Cluster three
**mPower MDS-UPDRS Part I**			
Mild	64.98	67.00	58.33
Moderate	24.24	21.96	22.50
Severe	10.78	11.04	19.17
**mPower MDS-UPDRS Part II**			
Mild	73.06	76.25	68.05
Moderate	26.94	23.63	31.95
Severe	0.00	0.12	0.00

**Table 3:** Statistical analysis for the three clusters.

	Cluster one	Cluster two	Cluster three
corXY	$- - -$	+ + +	+ + +
numberTaps	+ + +	+ + +	$- - -$
skewDriftRight	N.S.	+ +	$- - -$
skewDriftLeft	N.S.	+ +	$- - -$
cvTapInter	N.S.	+ + +	$- - -$
cvDriftRight	N.S.	N.S.	$- -$
cvDriftLeft	N.S.	N.S.	$- - -$
meanTapInter	$- - -$	$- - -$	+ + +
medianTapInter	$- - -$	$- -$	+ + +

The plus symbol (+) means the population average in a cluster is higher than the average of the baseline while the minus symbol (−) means that it is lower. N.S. means there is no statistical difference. The number of markers means the *p* value is less than 0.05, 0.01, and 0.001, respectively.

For statistical analysis of the clustering result, the one-sample t-test was used to determine the characteristics of clusters. One-sample t-test is a statistical hypothesis testing for determining whether the sample mean is statistically different from a known or hypothesized mean of the population^39^. The hypothesis for this study is *the population average in a cluster is equal to the overall participants average for each grouped feature (baseline)*. To adjust the significance level of hypothesis testing, we also apply Bonferroni correction and set the parameter of the family-wise error to 0.05^40^. Table 3 shows the attributes of the three clusters based on the hypothesis results.

The characteristics of each cluster are described as follow:

- *Cluster One* This cluster has the lowest severity of PD. This group has the feature values of numberTaps significantly higher than the baseline, while the feature values of corXY, meanTapInter, and medianTapInter are significantly lower than the baseline.
- *Cluster Two* This cluster has the second lowest severity of PD. This group has the feature values of corXY, numberTaps, skewDriftRight, skewDriftLeft, and cvTapInter significantly higher than the baseline, while the feature values of cmeanTapInter, and medianTapInter are significantly lower than the baseline.
- *Cluster Three* This cluster has the highest severity of PD. This group has the feature values of corXY, meanTapInter, and medianTapInter significantly higher than the baseline, and other features lower than the baseline.

There are a few research teams that published recent works on the mobile PD severity classification. Zhan et al.^41^ proposed a model to quantify PD motor symptom severity based on five tasks on a smartphone application. The tasks consist of voice recording, finger tapping, gait, balance, and reaction time. The scoring model was derived from 129 participants with 23 PD patients and 28 control participants who completed an in-clinic assessment and found to be correlated well with the MDS-UPDRS (r = 0.81; $p < .001$) and the Hoehn and Yahr stage (r = 0.91; $p < .001$). Sano et al.^42^ proposed an index for quantifying the severity of symptoms related to the finger-tapping of PD patients based on the 21 features extracted from the finger-tapping waveforms from magnetic sensors. The index gave a mean square error of 0.45 against the finger-tapping part of the UPDRS scored by a doctor. Therefore, they concluded that the index had a high correlation when validated with 31 PD patients and 360 control participants. While Zhan et al. reported the possibility of the severity assessment via a smartphone and Sano et al. proposed an index for quantifying the severity based on magnetic sensors, our results validated on a large scale that PD severity assessment could be done remotely by a personal smartphone without using any sensitive information.

### Limitation

In our study, data on clinical severity measurement was limited to parts I and II of MDS-UPDRS, not a full rating scale that comprises four parts. While this assessment is potentially a limitation, the relationship between health-related quality of life (HRQoL) and MDS-UPDRS was only demonstrated in MDS-UPDRS parts I and II, not parts III and IV, in a large multicentre study involving more than 3000 PD patients^43^. Moreover, the five items most significantly associated with PDQ8 were depressed mood (1.3), dressing (2.5), apathy (1.5), pain (1.9), and fatigue (1.13), all representing items within MDS-UPDRS Parts I and II. In terms of HRQoL assessment, both the PDQ-39 and the PDQ-8 were both included as ‘recommended’ scales in patients with PD. Whereas the PDQ-39 is the most thoroughly tested and applied questionnaire, the PDQ-8 has the advantages of being shorter and simpler to perform^44^. In a longitudinal study of over 1800 PD patients, the PDQ-8 was found to closely replicate results obtained from the parent form, PDQ-39, providing reliable and accurate information of HRQoL in PD patients^45^. In addition, the PDQ-8 has been evaluated in PD patients of different cultural backgrounds, including the Asian population^46^.

Although, the clusters were able to discriminate the severity of both non-motor and motor symptoms, as illustrated by the percentage of the participants in each cluster. Since there are fewer participants in the moderate and severe groups for Part II, the average scores for all three clusters were assigned to the mild group. Finger tapping is just one part of the motor symptoms. It might not be able to represent motor symptoms severity as a whole. However, finger tapping represents a feature of bradykinesia that could also affect the quality of life. Nevertheless, our analysis of the finger tapping features suggested that it could be one of the tools for quantification of PD severity outside of a clinical environment. The current aim of this study is to explore a relationship between tapping activity and PD severity from a large dataset. In the first stage, we examined how the tapping activity can be clustered and the relationship between such clusters and PD severity. Hence, we applied unsupervised machine learning, K-means clustering, to separate the patients based on the tapping activity data. The insight from the analysis in this study could aid with the creation of a more accurate supervised classification model for PD severity based on the finger tapping data, which is the plan for our future investigation.

## Conclusion

In this study, we performed an unsupervised clustering based on tapping features. Tapping activities were recorded on the participant’s mobile device. Based on the recording, tapping features were extracted. Nine of the extracted features were used for the clustering. For tapping activity and MDS-UPDRS I–II, there are a total of 1851 samples for the analysis; 1061 of those samples also contained PDQ-8^15^. From the results of evaluation metrics and expert opinion, we decide that three clusters was appropriate for our study. Each group has different characteristics based on tapping features and could represent different PD severity based on the MDS-UPDRS and PDQ-8 scores. Our study provides another evidence that quantitative measurement in a form of finger tapping can complement standard rating scales like MDS-UPDRS for characterizing PD heterogeneity^47^. Furthermore, since the model does not require sensitive information, the approach could pave the way to the remote anonymous screening and diagnosis in the future. We believe that any additional methods that could potentially assist with quantitative assessment of disease severity, without the need for a clinical visit would be beneficial to both the healthcare professionals and patients. One of the limitations of this study is that the data on clinical severity measurement was limited to parts I and II of MDS-UPDRS. However, the insight from the unsupervised analysis in this study could aid with the creation of a more accurate supervised classification model for PD severity based on the finger tapping data. Further research on the verification of the results with other datasets or the combination with other features such as gait and memory could also be explored.

## Data Availability

These data were contributed by users of the Parkinson mPower mobile application as part of the mPower study developed by Sage Bionetworks and described in Synapse [https://www.synapse.org/mPower]. The preprocessed datasets generated for this study are available on request to the corresponding author.
